# Continuous Serratus - Intercostal Plane Block for Perioperative Analgesia in Upper Abdominal Surgeries: A Prospective Randomized Controlled Study

**DOI:** 10.4274/TJAR.2023.231260

**Published:** 2023-10-24

**Authors:** Mohamed A Mamoun, Alrefaey K. Alrefaey, Maha Ahmed Abo-Zeid

**Affiliations:** 1 Department of Anaesthesia and Surgical Intensive Care, Mansoura University, Mansoura, Egypt

**Keywords:** Abdominal surgery, analgesia, block, pain, regional anaesthesia

## Abstract

**Objective:**

Acute pain management after open abdominal surgeries is an essential goal in perioperative management.. Recently, serratus-intercostal plane block (SIPB) was suggested as an analgesic technique for upper abdominal surgeries.

**Methods:**

This prospective, randomized, controlled study included sixty adult patients scheduled for open upper abdominal surgeries. Patients were allocated into two equal groups: SIPB group (S group, n = 30) and control group (the C group, n = 30). In the S group, SIPB was performed in the midaxillary line at the eighth rib level followed by continuous infusion of local anaesthetic for the first postoperative day. In the C group, no block was done. The primary objective of the study was to control postoperative pain on the first postoperative day as assessed by the numerical rating scale (NRS). Secondary outcomes included perioperative hemodynamics, total postoperative analgesic consumption, number of analgesic requests, and incidence of postoperative nausea and vomiting.

**Results:**

The mean postoperative NRS reported in group S was statistically lower than that in group C (2.4±0.7, 3.9±0.31, *P* < 0.001). The postoperative morphine consumption was lower in the S group than in the C group [(0 (0-4), 3 (1-4), respectively, *P* < 0.001]. The incidence of PONV was significantly lower in the S group than in the C group (16.7% and 40%, *P* < 0.045).

**Conclusion:**

SIPB was associated with a better analgesic profile compared with the control group after upper abdominal surgeries. Further studies are recommended to determine block safety in special patient groups, including bariatric and laparoscopic surgeries.

**Main Points**

• Upper abdominal incisions are associated with substantial postoperative pain.

• Technical difficulties, hemodynamic effects, and contraindications in certain groups of patients limit the use of most regional techniques.

• Recently, the serratus-intercostal plane block (SIPB) was reported as an effective analgesic technique for upper abdominal surgeries.

• This study investigated the perioperative analgesic effect of continuous SIPB plane block with the hypothesis that the block will offer an adequate analgesic option for patients undergoing elective open abdominal surgeries.

## Introduction

Upper abdominal incisions are associated with a substantial degree of postoperative pain, which is associated with higher rates of postoperative complications, delayed ambulation, and prolonged hospital stay. Diverse analgesic modalities have been used to control pain after abdominal surgeries; including neuraxial techniques, abdominal wall block, and systemic analgesics.^1,2^

The epidural technique, which is the gold standard analgesia technique for abdominal surgeries, is limited by technical difficulties, hemodynamic effects, and contraindications in certain groups of patients.^3^ As an alternative, abdominal wall blocks have been used for different open and laparoscopic abdominal surgeries; however, the ambiguity of the sonographic view in obese patients and the fear of iatrogenic injury to the viscera could complicate the procedure.^4^

Recently, few studies have reported serratus-intercostal plane block (SIPB) as an effective analgesic technique for upper abdominal surgeries. The diffusion of local anaesthetic (LA) in this fascial plane blocks the cutaneous branches of the 7^th^ to the 11^th^ intercostal nerves, providing adequate analgesia for the control of pain after upper abdominal surgeries.^5^ The suggested advantages of SIPB include the ease of the technique, the solid landmarks, and the possible  administration in the supine position.^6^

In this study, continuous SIPB; using catheter technique was investigated as an adequate analgesic option for patients undergoing elective open abdominal surgeries. The primary objective of the study was to control postoperative pain assessed by the numerical rating scale (NRS) on the first postoperative day. Secondary outcomes included perioperative hemodynamics, postoperative analgesic consumption, number of postoperative analgesic requests, and postoperative nausea and vomiting (PONV).

## Methods

This prospective, randomized, controlled study was conducted after Medical Research Ethics Committee, Institutional Review Board of Mansoura University approval (approval no: R.21.02.1217, date: 03.04.2021) and clinical trial registry (PACTR202105543447214). Informed consent was obtained from eligible candidates, including all adult patients of American Society of Anaesthesiologists physical status I and II, who were scheduled for open abdominal surgeries through a unilateral incision. Patients with chest wall deformities, neuromuscular disease, and a known allergy to the study medications were excluded from the study.

### Randomization

A randomization list, in blocks of 10, was used to allocate patients into two equal groups (Sealed Envelope.com, Seed no. 64866366541202 )^7^; SIPB Group (S group, n = 30) and control group (C group, n = 30). All patients were subjected to routine preoperative assessment, including medical history, physical examination, and laboratory investigations, and additional assessment was performed as per attending anaesthesiologist’s recommendations. All included patients were educated about the use of a 10-mm NRS for pain assessment (0 mm= no pain, and 10= the worst possible pain).

### General Anaesthesia

Upon arrival to the operative room, patients were monitored using basic monitors [heart rate (HR), non-invasive arterial blood pressure, and peripheral oxygen saturation]. General anaesthesia was induced by intravenous (IV) administration of 2 µ kg^-1^ of fentanyl, 1-2 mg kg^-1^ of propofol, and 0.5 mg kg^-1^ of atracurium. A properly sized endotracheal tube was inserted and fixed in place after confirmation of correct positioning. Anaesthesia was maintained using sevoflurane in 40% oxygen-air mixture.

### Block Procedure

**Figure 1:**
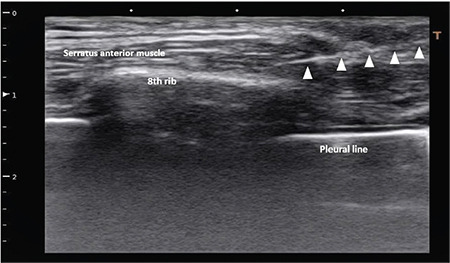
Ultrasound view for the SIPB block, showing the 8^th^ rib with the covering Serratus muscle layer, and the tip of the catheter floating in the local anaesthesia bolus. SIPB, serratus-intercostal plane block.

After ensuring patient stability, the study protocol was applied according to the allocation sequence. In the S group, under aseptic conditions, with the patient lying supine, the linear probe (Toshiba Xario, Japan, PLT 805AT transducer) was placed sagittally at the 8^th^ rib in the midaxillary line. An 80-mm Tuohy needle was introduced via an in-plane approach until the needle tip was adjacent to the 8^th^ rib between the serratus and external intercostal muscles. A test for correct placement of the needle was performed using 2-3 mL of normal saline, and then 20 mL of 0.25% bupivacaine was injected between the external intercostal and the serratus anterior muscle after negative blood aspiration. After bolus injection, a 20-gauge catheter (Perifix® Epidural anaesthesia catheter, Braun Medical Inc., USA) was inserted into the interfacial plane and fixed in place using adhesive tape (Figure 1).

### Perioperative Analgesia

After catheter insertion and fixation, a continuous infusion of 0.125% bupivacaine was initiated, at a rate of 4 mL h, through the catheter using an elastomeric infusion pump

(Disposable infusion pump, Zhejiang Fert medical device Co., China- 100 mL capacity, 0.5 mL bolus, lock time 15 minutes), with no injection was performed in C group.

For both groups, IV 50 µg fentanyl bolus was administered when intraoperative rescue analgesia was deemed indicated by the attending anaesthetist or if the hemodynamic measurements increased by >20% of the basal recording (in the absence of other causes). Additionally, 1 g of acetaminophen was given 30-minutes before skin closure in both groups. After skin closure, the patient was extubated upon fulfilling the extubation criteria and adequate reversal of the muscle relaxant. The postoperative analgesia order included regular IV paracetamol (1 g/6 h) administered to all patients. If postoperative NRS ≥4, a rescue dose of IV morphine (0.02 mg kg^-1^) was administered. Patients were admitted to a high dependency unit for 24 h monitoring after surgery. Thereafter, the catheter was removed and its site was inspected for any sign of edema, redness, hematoma, or discharge.

### Outcomes

The collected data included patient age, weight, height, gender, type of surgery, perioperative hemodynamics (HR, MAP) measured at induction, 15 min, 30 min, and hourly until end of surgery, analgesic bolus requirements, NRS (at 0, 2, 4, 8, 12, and 24 h after surgery), analgesic bolus requirements, incidence of PONV, and any catheter site complications. Data were collected and recorded by an independent nurse trained in the study protocol.

### Sample Size Calculation and Statistical Analysis

Depending on the results of a previous study,^8^ the mean postoperative pain score after upper abdominal surgeries was (NRS 4.8±1.6). Adopting a 25% reduction of the mean pain score after SIPB as an accepted effect size to attain a study power of 0.80 with an alpha error of 0.05; 27 patients per group were required. Adapting for 10% dropout, 30 patients were sufficient. G*power software version 3.1.9.7 was used for the sample size calculation.

IBM SPSS (USA) version 22 was used for statistical analysis of the collected data. Data were explored for normality of distribution and presented as mean ± standard deviation, median (minimum-maximum), or number (percentage). Statistical differences between the two studied groups were analyzed using the independent samples t-test, Mann-Whitney test, or chi-square test as appropriate. *P* value was considered significant if less than 0.05.

## Results

**Figure 2:**
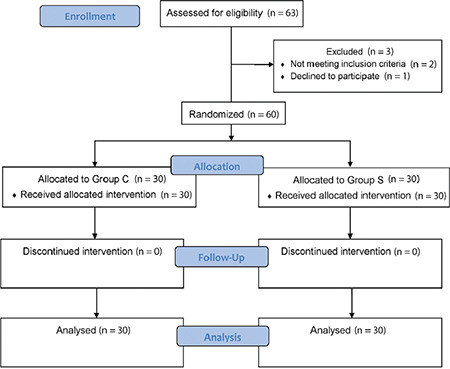
Study consort chart.

In this study, 63 patients scheduled for open upper abdominal surgery through a unilateral incision were recruited from the study from May 2021 to July 2022. As shown in Figure 2, three patients were excluded from the study. The included patients were randomly divided into two groups: 30 patients each.

**Figure 3:**
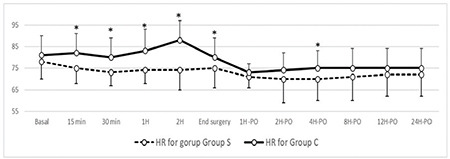
Perioperative heart rate in the two studied groups. *Indicates *P* value of less than 0.05. Bpm, beat per minute; HR, heart rate.

**Figure 4:**
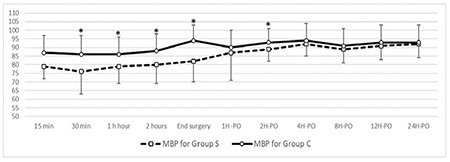
Perioperative mean arterial pressure in the two studied groups. *Indicates *P* value of less than 0.05. MBP, mean blood pressure.

Patient basal data and perioperative characteristics, duration, incision type, and type of surgery were comparable between the two groups (Table 1). Perioperative hemodynamics are shown in Figures 3 and 4. Intraoperative hemodynamics [HR and mean blood pressure (MBP)] showed statistically significant lower readings in group S. Postoperative hemodynamic readings continued to be lower in group S despite could not reach statistical significance except for the MBP reading for the second postoperative hour.

Table 2 shows the intraoperative and postoperative analgesic profiles for the two studied groups. The number of intraoperative analgesic boluses was statistically lower in group S [0 (0-1), 1 (0-2), *P < 0.001*]. Postoperatively, the reported NRS was statistically lower in group S than in group C at 0, 2, 4, 8 hours after surgery. Likewise, a statistically significant reduction in both the mean postoperative NRS value and the total postoperative morphine consumption (mg) was noticed lower in group S than in group C [(2.4±0.7 vs. 3.9±0.31, *P < 0.001*, 0 (0-4) 3 (1.4-4.1), *P < 0.001*, respectively]. In addition, Table 2 shows a statistically lower incidence of PONV in group S than in group C (16.7% and 40%, *P* 0.045 respectively).

None of the cases in group S showed any edema, redness, hematoma, or discharge at the site of catheter insertion.

## Discussion

In this study, continuous SIPB was used for perioperative analgesia open abdominal surgeries through a unilateral incision. Compared with IV opioid, SIPB showed a better analgesia profile, less postoperative morphine consumption and decreased incidence of PONV. To the best of our knowledge, this is the first study to apply continuous SIPB for analgesia after unilateral abdominal incisions.

Acute pain management after open abdominal surgeries is an essential goal in perioperative management, especially in the era of enhanced recovery programs.^9,10^ Inadequate analgesia is associated with higher rates of postoperative complications, delayed ambulation, and prolonged hospital stay.^11^

Analgesic practices for abdominal incisions include a wide range of techniques. IV analgesics either bolus or continuous infusion were associated with respiratory depression, delayed intestinal motility, and PONV. Opioid crisis worldwide have triggered the use of opioid-saving strategies, including neuraxial, regional, and facial plane blocks.^1,8,10,12,13,14,15^

Neuraxial analgesic techniques, including intrathecal, epidural and paravertebral blocks, have proven to have great analgesic efficacy. Nevertheless, neuraxial procedures are considered technically difficult, and they also carry some risk in special groups of patients, e.g., stenotic valve diseaser receiving anticoagulants.^16,17^

Facial plane blocks, such as transversus abdominal plane (TAP) and rectus sheath blocks, have been proven to decrease pain scores after abdominal surgeries, especially with the evolution of ultrasound technology. However, difficulties are usually encountered in obese patients and in patients with previous incisions that obscure the proper visualization of the interfacial planes or are even disturbing. In addition, preemptive use of TAP block can cause escape of LA outside the targeted space after surgical incision. In addition, serious complications such as vascular injury, abdominal visceral injury, liver trauma, and nerve injuries have been reported.^18,19,20^

The first description of SIPB was in 2013^21^ using the acronym BRILMA block (Blocking the branches of intercostal nerves in the middle axillary line). Advancement of the technique follows, with subsequent modification to target the lower intercostal nerves at the level of the 8^th^ rib; modified BRILMA block.  The block was effective for somatic analgesia after gastrectomy and cholecystectomy in a small series of cases.^21,22^ Afterwards, more than one study reported the effectiveness of SIPB for open upper abdominal surgeries.^5,8,23^ SIPB lacks an analgesic effect on visceral pain, yet control of the somatic component of postoperative pain can result in satisfactory pain scores and minimize the required rescue analgesics.

Several studies have used the SIPB bolus technique for analgesia after unilateral abdominal surgeries.^5,8,21,22,24^ Compared with the oblique subcostal TAB block; SIPB compared with the rectus sheath block significantly improved the quality of analgesia and lowered the analgesic requirement in patients undergoing laparoscopic cholecystectomy.^5^ Interestingly, bilateral SIPB was effectively used as a rescue analgesia after bariatric surgery, achieving an adequate NRS pain score after 10 min and lasting for 46 hours.^24^

In a prospective randomized study, 102 patients were divided into a control group receiving IV morphine and an interventional group receiving SIPB. The SIPB group showed lower pain scores, lower postoperative opioid consumption and improved quality of recovery (QoR-15 scores) 24 h postoperatively.^23^

PONV is an important patient outcome that was recorded in our study and was found to be significantly lower in patients who received SIPB. This can be explained by the lower postoperative opioid consumption, which is included in the Apfel score as one of the risk factors for PONV.^25^ Lower PONV can increase patient satisfaction and shorten the in-hospital length of stay.^26^

### Study Limitations

Limitations of the study included the heterogeneous group of patients with different surgical approaches and incisions and variable pain profiles. However, this can support the wide applicability and effectiveness of the SIPB. Secondly, the use of an elastomeric pump, which has a fixed preset infusion rate, should be used in all patients, rather than individualized. Nevertheless, simplicity, non-electricity, and better patient mobilization motivated the authors to use an elastomeric pump instead of electricity-driven syringe pumps. Assessment of dermatomal distribution of analgesia was limited by the surgical wound and dressing. The serum level of LA was not measured in our study, the LA concentration reached after bolus and continuous infusion in SIPB can assess the degree of systemic absorption, verify block safety, and help determine the mechanism of action of the block. A double-blind protocol could avoid data collection bias and the placebo effect; nevertheless, this could not be achieved in this study because of the nature of the intervention.

## Conclusion

Continuous SIPB can be added to the arsenal of analgesic techniques used for analgesia after abdominal surgeries with a unilateral incision. In our study, SIPB was associated with a better analgesic profile and lower analgesic consumption. Further studies are recommended to determine block safety in special patient groups, including bariatric and laparoscopic surgeries.

## Figures and Tables

**Table 1:**
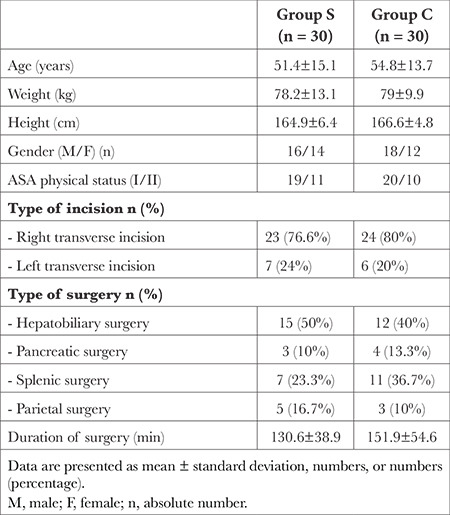
Patients' Basal Characteristics, Duration and Type of Surgery in the Two Studied Groups

**Table 2:**
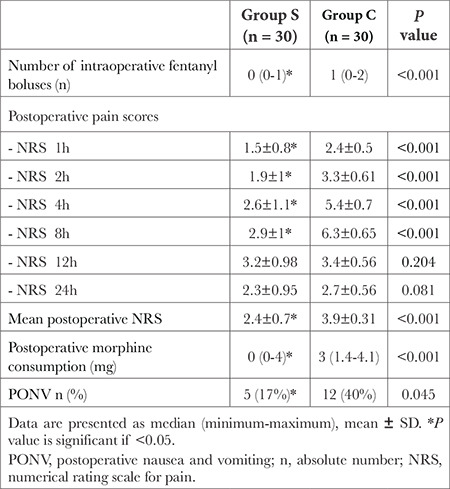
Analgesic Profile and the Incidence of PONV in the Two Studied Groups
